# A Genetic Score Associates With Pioglitazone Response in Patients With Non-alcoholic Steatohepatitis

**DOI:** 10.3389/fphar.2018.00752

**Published:** 2018-07-17

**Authors:** Marina Kawaguchi-Suzuki, Kenneth Cusi, Fernando Bril, Yan Gong, Taimour Langaee, Reginald F. Frye

**Affiliations:** ^1^Department of Pharmacotherapy and Translational Research, University of Florida, Gainesville, FL, United States; ^2^Center for Pharmacogenomics, University of Florida, Gainesville, FL, United States; ^3^Pacific University School of Pharmacy, Hillsboro, OR, United States; ^4^Division of Endocrinology, Diabetes & Metabolism, University of Florida, Gainesville, FL, United States; ^5^Endocrinology, Diabetes and Metabolism, Malcom Randall VA Medical Center, Gainesville, FL, United States

**Keywords:** NAFLD, fatty liver, NASH, thiazolidinediones, pharmacogenetics

## Abstract

Pioglitazone is used effectively to treat non-alcoholic steatohepatitis (NASH), but there is marked variability in response. This study examined whether genetic variation contributes to pioglitazone response variability in patients with NASH. This genetic substudy includes 55 participants of a randomized controlled trial designed to determine the efficacy of long-term pioglitazone treatment in patients with NASH. The primary outcome of the clinical trial was defined as ≥2-point reduction in the non-alcoholic fatty liver disease activity score (NAS). In this substudy, single nucleotide polymorphisms (SNPs) in putative candidate genes were tested for association with primary and secondary outcomes. A genetic response score was constructed based on the sum of response alleles for selected genes. The genetic response score was significantly associated with achievement of the primary outcome (odds ratio 1.74; 95% CI 1.27–2.54; *p* = 0.0015). *ADORA1* rs903361 associated with resolution of NASH (*p* = 0.0005) and change in the ballooning score among Caucasian and Hispanic patients (*p* = 0.0005). *LPL* rs10099160 was significantly associated with change in ALT (*p* = 0.0005). The *CYP2C8^∗^3* allele, which confers faster pioglitazone clearance in allele carriers, was associated with change in fibrosis score (*p* = 0.026). This study identified key genetic factors that explain some of the inter-individual variability in response to pioglitazone among patients with NASH.

## Introduction

Clinical guidelines support the use of pioglitazone as a pharmacological agent to manage biopsy-proven non-alcoholic steatohepatitis or NASH (Chalasani et al., 2017). Pioglitazone is a PPAR-γ agonist that belongs to the thiazolidinedione (TZD) class of antidiabetic drugs (Cariou et al., 2012). PPAR-γ is highly expressed in adipose tissue, and TZDs are thought to work primarily through PPAR-γ in adipocytes (Cariou et al., 2012). However, PPAR-γ is also expressed in non-parenchymal cells such as Kupffer and stellate cells, and parenchymal cell expression is induced in NAFLD (Boelsterli and Bedoucha, 2002; Westerbacka et al., 2007; Pettinelli and Videla, 2011). Additionally, PPAR-γ was shown to modulate the production and secretion of various adipokines, such as adiponectin, resistin, tumor necrosis factor-α, and monocyte chemoattractant protein-1 (Cariou et al., 2012). Although the exact mechanism is not clear, the immune-modulatory and anti-inflammatory effects of PPAR-γ may also play a role in NAFLD (Cariou et al., 2012).

According to a meta-analysis of randomized clinical trials, pioglitazone significantly improved hepatic steatosis (OR 4.05; 95% CI 2.58–6.35) and inflammation (OR 3.53; 95% CI 2.21–5.64) (Vernon et al., 2011). Another meta-analysis showed that pioglitazone treatment was associated with improvement in advanced fibrosis (OR, 2.95; 95% CI, 1.04–10.90) and NASH resolution (OR, 3.40; 95% CI, 1.95–5.93); notably, advanced fibrosis also improved with pioglitazone treatment in non-diabetic patients with NASH (Musso et al., 2017). Thus, clinical evidence supports use of pioglitazone as a potentially effective agent to treat NASH. However, significant inter-patient variability in response to this drug has been observed. In the PIVENS study, one of the largest clinical trials investigating the use of pioglitazone in NASH, pioglitazone was shown to improve liver histology scores compared to placebo, but only 34% of patients randomized to the pioglitazone arm demonstrated improvement in the primary outcome (Sanyal et al., 2010).

The extent to which genetic factors contribute to this observed variability in pioglitazone response is not known. Genetic predisposition to NAFLD and NASH has been described previously, and the estimated heritability of NAFLD is approximately 39% (Schwimmer et al., 2009; Cohen et al., 2011; Dongiovanni et al., 2013). A genetic risk for development of NASH and fibrosis has been demonstrated, and two genome-wide association studies have shown an association of *PNPLA3* polymorphisms (rs738409 and rs2281135) with liver-enzyme levels and susceptibility to NAFLD (Romeo et al., 2008; Yuan et al., 2008; Sookoian et al., 2009; Valenti et al., 2010; Cohen et al., 2011; Hernaez et al., 2013). Although the genetic contribution to disease susceptibility has been examined, it is not known whether the drug response in patients with NASH can be explained by genetic factors. Among healthy volunteers and diabetic patients, various genetic polymorphisms were determined to influence both pioglitazone pharmacokinetics and pharmacodynamics (Kawaguchi-Suzuki and Frye, 2013). For example, the *CYP2C8* gene codes for CYP 2C8, the enzyme predominately responsible for the metabolism of pioglitazone (Eckland and Danhof, 2000); the *CYP2C8*^∗^3 allele, designated by the haplotype rs11572080 and rs10509681, results in reduced pioglitazone exposure due to faster drug clearance (Tornio et al., 2008; Aquilante et al., 2013; Kawaguchi-Suzuki and Frye, 2013). Multiple SNPs in genes related to the drug pathway were previously associated with pharmacodynamic response in patients with diabetes (Kawaguchi-Suzuki and Frye, 2013).

The purpose of this study was to investigate the effect of genetic polymorphisms on the liver histological response to pioglitazone treatment in patients with NASH.

## Patients and Methods

### Study Participants and Design

As part of a randomized, double-blind, placebo-controlled trial to evaluate the efficacy and safety of long-term pioglitazone treatment in patients with NASH (ClinicalTrials.gov: NCT00994682), 101 patients were recruited from the general population of San Antonio, Texas, between December 2008 and 2014. Complete inclusion/exclusion criteria and results from the main study have been reported previously (Hjelkrem et al., 2011; Cusi et al., 2016). Briefly, the study participants were aged between 18 and 70 years and had prediabetes or type 2 diabetes and biopsy-proven NASH. The participants were prescribed a hypocaloric diet (−500 kcal/day) by a research dietician and were counseled on physical activity at baseline. Participants were randomly assigned in a 1:1 ratio to either pioglitazone (Actos^®^, Takeda Pharmaceuticals) 30 mg/day (titrated after 2 months to 45 mg/day) or placebo. The primary outcome was a reduction of at least two points in the NAS, with improvement in at least two different categories and without worsening of fibrosis after 18 months of therapy. Secondary liver histologic outcomes included resolution of NASH and improvement in individual histologic scores. In addition, concentration changes in the laboratory biomarkers, plasma alanine aminotransferase (ALT), aspartate aminotransferase (AST), and CK-18, were assessed to determine if significant changes were observed after pioglitazone treatment.

After 18 months of treatment with pioglitazone, liver biopsies were repeated and participants were offered to continue for another 18 months with open-label pioglitazone treatment. Participants had follow-up visits every 2 months, and liver biopsies were repeated at the end of the open-label phase. In this genetic substudy, we report on 55 participants of the randomized controlled trial who had blood samples available – 32 participants from the blinded randomized phase and 23 participants from the open-label phase (Cusi et al., 2016). This study was carried out in accordance with the recommendations of the University of Texas Health Science Center Institutional Review Board. The research protocol was approved by the local institutional review board, and all participants gave written informed consent prior to participation in accordance with the Declaration of Helsinki.

### Genotyping

For each study participant, DNA was extracted from blood samples using the FlexiGene DNA kit (QUIAGEN Inc, Valencia, CA, United States). Customized chips were used to genotype for 60 SNPs with QuantStudio 12K Flex Real-Time PCR System (Thermo Fisher Scientific Inc, Waltham, MA, United States) (Supplementary Table S1).

The genes and SNPs were selected based on previous association with pharmacokinetics or pharmacodynamics of pioglitazone or rosiglitazone in healthy volunteers or diabetic patients (Kawaguchi-Suzuki and Frye, 2013). Additionally, tagging SNPs were selected for *PPARG*, *ADIPOQ*, *LPL*, and *RETN* genes with HaploView (Broad Institute, Cambridge, MA, United States) with *r*^2^ > 0.8 and MAF of >0.1 (*PPARG*), >0.15 (*ADIPOQ* and *LPL*), and >0.25 (*RETN*) in CEU and MEX populations (Barrett et al., 2005). All DNA samples were analyzed using two separate chips to confirm matching genotypes. Participants or SNPs were excluded if call rates were below 95%.

### Statistical Methods

Paired *t*-tests were performed to compare biopsy scores and biomarker levels at baseline and after treatment. Logistic regression was performed for binary traits such as the primary outcome (yes/no) and linear regression was performed for continuous traits, such as concentration change in laboratory biomarkers. SNP-trait association analyses were conducted separately for each race/ethnicity by adjusting for age, gender, and respective baseline histology score or biomarker level. Then, meta-analysis was performed to obtain the summary effects of the entire study population. Heterogeneity among different race/ethnicity groups were assessed using inconsistency index (I^2^) and Cochran’s *Q*. When there was no evidence of heterogeneity (I^2^ < 50% and Cochran’s *Q* *p*-value > 0.1), an inverse variance fixed-effect model was used for the meta-analyses to combine the effect of the three race/ethnicity groups. The SNPs with *p*-value < 0.0008 were considered statistically significant to account for the multiple comparisons of the 60 SNPs based on Bonferroni-correction. The SNPs with *p* < 0.05 were considered nominally significant. A power calculation indicated that at alpha of 0.0008, we have 80% power to detect OR of ≥5.3 for SNPs with MAF of 30% or higher.

A genotype score was constructed by summing the number of protective alleles to estimate the summarized effect of nominally significant SNPs for the primary outcome. Regarding the achievement of the primary outcome or resolution of NASH, the Cochran Armitage trend test was used to analyze the effect of each allele for the nominally significant SNPs. Logistic regression was used to assess the effect of the calculated score on the primary outcome. Regarding changes in biopsy scores, univariate analyses was conducted with Tukey’s multiple comparisons test to examine difference between genotypes of the nominally significant SNPs. The genetic score was considered significant if *p*-value < 0.05. Sensitivity analysis was conducted in the subset of patients excluding the smallest race/ethnicity group (i.e., “other” race/ethnicity) to confirm the identified SNPs. Hardy–Weinberg equilibrium was assessed with a χ^2^ test with one degree of freedom. All analyses were performed in JMP Genomics version 6.0 (SAS Institute Inc., Cary, NC, United States).

## Results

Baseline characteristics of participants are summarized in **Table 1**. These characteristics were similar to the entire cohort of participants enrolled in the main randomized controlled trial, as previously reported (Cusi et al., 2016). Plasma AST and ALT concentration, as well as plasma CK-18 concentration, decreased significantly with pioglitazone treatment compared to baseline levels (**Table 2**). The primary outcome was achieved in 32 (58%) of the genetic substudy participants. Additionally, secondary outcomes were significantly improved with the TZD. For example, among the 46 participants who had a definite diagnosis of NASH at baseline, 28 (60%) participants had resolution of NASH with pioglitazone. The NAS and individual histologic parameters were all improved significantly (**Table 2**). All genotypes were confirmed with matching results from two different chips. All participants had sample call rates > 98.3% and all SNPs had genotype call rate > 99.5%; none of the participants or SNPs were excluded. None of the genotype frequencies of the SNPs significantly deviated from Hardy-Weinberg equilibrium.

**Table 1:** Baseline patient characteristics.

Characteristic	Baseline (*N* = 55)
Age (years)	54 ± 9
Gender (male/female)	75%/25%
Race/ethnicity (Caucasian/Hispanic/other)	25%/64%/11%
Body mass index (kg/m^2^)	35 ± 5
Type 2 diabetes	60%
Metabolic syndrome	89%
Dyslipidemia	93%

*Values shown as mean ± standard deviation or percent.*

**Table 2:** Plasma aminotransferases, CK-18 and histologic changes after 18 months of pioglitazone treatment.

Phenotype	Baseline	After pioglitazone treatment	*P*-value
ALT (IU/L)	63 ± 41	30 ± 15	<0.0001
AST (IU/L)	50 ± 35	29 ± 10	<0.0001
CK-18 (IU/L)	356 ± 289	204 ± 164	0.001
NAS	4.6 ± 1.5	2.7 ± 1.7	<0.0001
Steatosis	2.1 ± 0.9	1.2 ± 0.9	<0.0001
Inflammation	1.7 ± 0.5	1.2 ± 0.7	<0.0001
Ballooning	0.9 ± 0.4	0.3 ± 0.5	<0.0001
Fibrosis	1.1 ± 1.1	0.8 ± 1.0	0.002

*Values shown as mean ± standard deviation.*

The results of the SNPs with nominal significance in the meta-analyses for primary outcome and change in NAS are summarized in **Table 3**. *PPARG* SNP rs4135275 and *LPL* SNP rs2197089 were nominally associated with higher odds for the primary outcome, while *LPL* SNPs rs253, rs10099160 and rs270 were nominally associated with lower risk for the primary outcome. A SNP summarized score was calculated based on the number of protective alleles to estimate the summarized effect of nominally significant SNPs for the primary outcome. This was based on SNPs *PPARG* rs4135275, *LPL* rs253, *LPL* rs10099160, *LPL* rs270, and *LPL* rs2197089 genotypes (Supplementary Table S2). The score values ranged from 1 to 10 with a median of 6 (IQR: 4–8). According to the logistic regression analysis, in univariate analysis the SNP summarized score was significantly associated with achievement of the primary outcome (OR 1.64, 95% CI 1.24–2.30; *p* = 0.0015, **Table 4**). In multivariable logistic regression analysis, taking into account baseline NAS, age, and gender, the results were nearly identical with an OR 1.74 (95% CI 1.27–2.54; *p* = 0.0015, **Table 4**).

**Table 3:** Single nucleotide polymorphism with a significant association with the primary outcome and/or change ≥ 2 in the NAFLD Activity Score^∗^.

Phenotype	SNP	Alleles	MAF	Protective Allele	OR or Beta	Lower 95% CI	Upper 95% CI	*P*-value
Primary	*PPARG* rs4135275	A/G	0.23	G	5.28	1.32	21.13	0.019
Outcome	*LPL* rs253	C/T	0.44	C	0.28	0.10	0.83	0.022
	*LPL* rs10099160	T/G	0.22	T	0.18	0.04	0.82	0.026
	*LPL* rs270	C/A	0.17	C	0.12	0.02	0.79	0.027
	*LPL* rs2197089	A/G	0.48	G	2.81	1.07	7.39	0.037
Change in	*RETN* rs4804765	G/T	0.38	T	−0.86	−1.43	−0.30	0.0028
NAS	*LPL* rs270	C/A	0.17	C	1.21	0.38	2.05	0.0045
	*LPL* rs13266204	A/G	0.15	A	0.96	0.27	1.66	0.0067
	*ABCA1* rs2230806	C/T	0.39	C	0.71	0.18	1.24	0.0086
	*LPL* rs10099160	T/G	0.22	T	0.90	0.15	1.64	0.018

*^∗^Alleles are listed as wild/variant alleles and from top to bottom in order of their significance for the respective outcome. Odds ratios are shown for the primary outcome, and beta values are listed for the change in NAS. All analyses were adjusted for age, gender, and baseline NAS. Minor allele frequencies by race/ethnicity are listed in Supplementary Table S3.*

**Table 4:** Summary of logistic regression for the primary outcome with the SNP summarized score based on *PPARG* rs4135275, *LPL* rs253, *LPL* rs10099160, *LPL* rs270, and *LPL* rs2197089 genotypes.

Parameter	OR	Lower 95% CI	Upper 95% CI	*P*-value
*Univariate analysis*				
SNP summarized score	1.64	1.24	2.30	0.0015
*Multivariate analysis*				
Age	0.99	0.92	1.08	0.8953
Gender	0.28	0.04	1.42	0.1467
Baseline NAS	1.69	1.08	2.91	0.0336
SNP summarized score	1.74	1.27	2.54	0.0015

**FIGURE 1:**
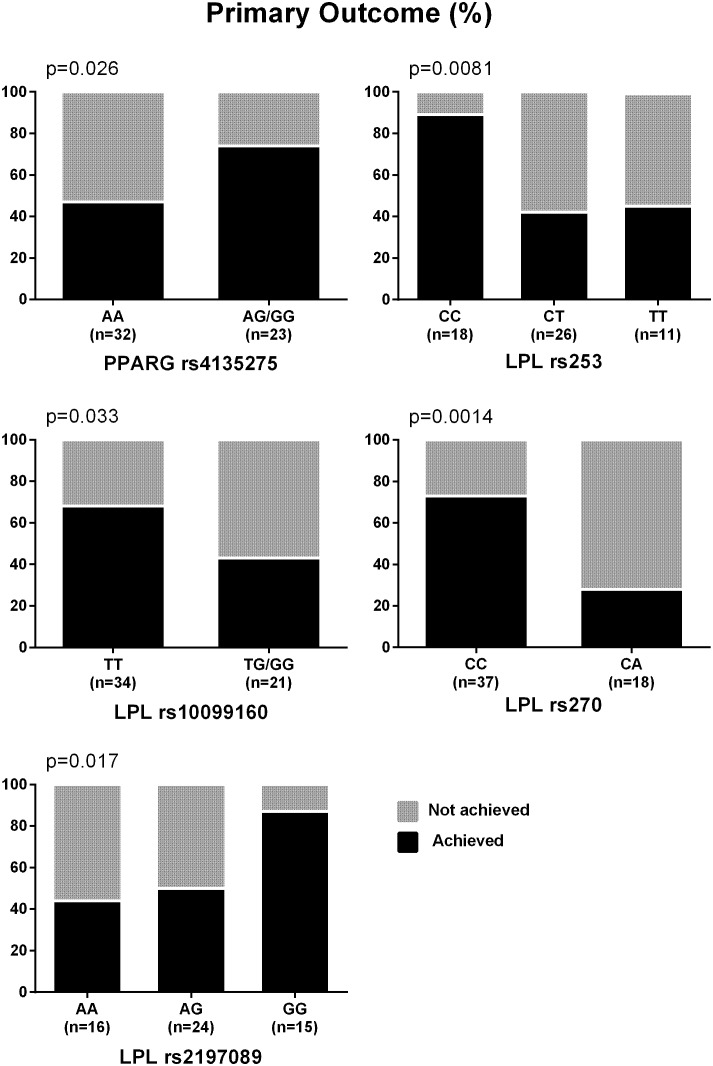
Percentages of participants who achieved the primary outcome. Primary outcome was defined as reduction in NAS ≥ 2 (at least one-point improvement in more than one liver histology category without any worsening of fibrosis). *P*-values from Cochran Armitage trend tests are shown.

**FIGURE 2:**
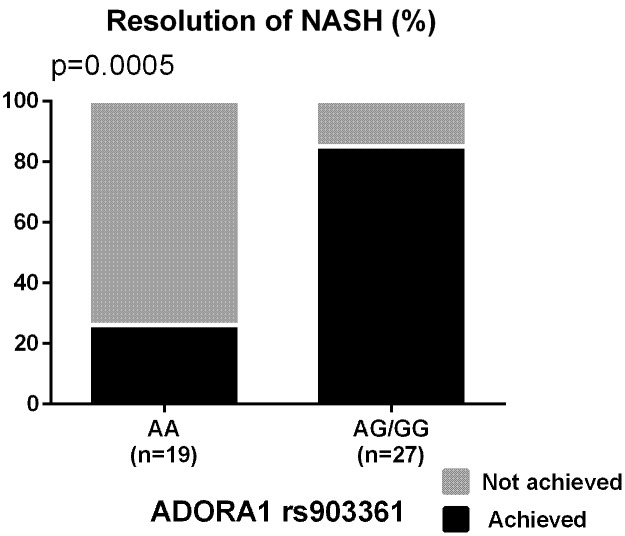
Percentages of participants who achieved the resolution of NASH based on *ADORA1* rs903361 genotype. *P*-values from Cochran Armitage trend tests are shown.

The percentages of participants who achieved the primary outcome based on the genotypes of the nominally significant SNPs identified by the meta-analyses are depicted in **Figure 1**. SNPs associated with NASH resolution were: *KCNQ1* rs2237895 (OR = 0.12, *p* = 0.012), *LPL* rs270 (OR = 0.07, *p* = 0.019), *ABCA1* rs2230806 (OR = 0.17, *p* = 0.019), *RETN* rs4804765 (OR = 4.41, *p* = 0.030), *ADORA1* rs903361 (OR = 5.54, *p* = 0.042). When the three race/ethnicity groups were evaluated together by the Cochran Armitage trend test, the presence of the *ADORA1* rs903361 G allele was significantly associated with NASH resolution (*p* = 0.0005, **Figure 2**).

When changes in AST/ALT, CK-18 and histology were examined as phenotypes, *LPL* rs10099160 met Bonferroni-corrected significance and was associated with change in plasma ALT (*p* = 0.00052). The *LPL* rs10099160 SNP was also associated with the primary outcome (*p* = 0.026) and change in NAS (*p* = 0.018). SNPs associated with change in steatosis score were: *ABCA1* rs2230806 (β = 0.35, *p* = 0.0087) and *RETN* rs4804765 (β = −0.32, *p* = 0.033). The only SNP associated with change in inflammation was *RETN* rs4804765 (β = −0.29, *p* = 0.0078). SNPs associated with change in ballooning were: *LPL* rs270 (β = 0.36, *p* = 0.0048), *LPL* rs253 (β = 0.23, *p* = 0.0057), *KCNQ1* rs2237895 (β = 0.22, *p* = 0.0084), *LPL* rs13266204 (β = 0.27, *p* = 0.011), *ABCA1* rs2230806 (β = 0.18, *p* = 0.031), and *RETN* rs4804765 (β = −0.19, *p* = 0.034). *CYP2C8* rs11572080 (β = 0.61, *p* = 0.0056), *LPIN1* rs10192566 (β = −0.31, *p* = 0.0095), *ADIPOQ* rs266729 (β = −0.35, *p* = 0.022), and *ADIPOQ* rs182052 (β = −0.25, *p* = 0.044) were associated with changes in fibrosis.

In addition to the *LPL* rs10099160, some of the SNPs demonstrated consistent association among different phenotypes. SNPs showing consistent association were: *ABCA1* rs2230806, *KCNQ1* rs2237895, *LPL* rs253, *LPL* rs270, *LPL* rs13266204, and *RETN* rs4804765. *LPL* rs270 also showed nominal significance when the association was assessed with change in plasma ALT level (β = 8.84, *p* = 0.01).

**FIGURE 3:**
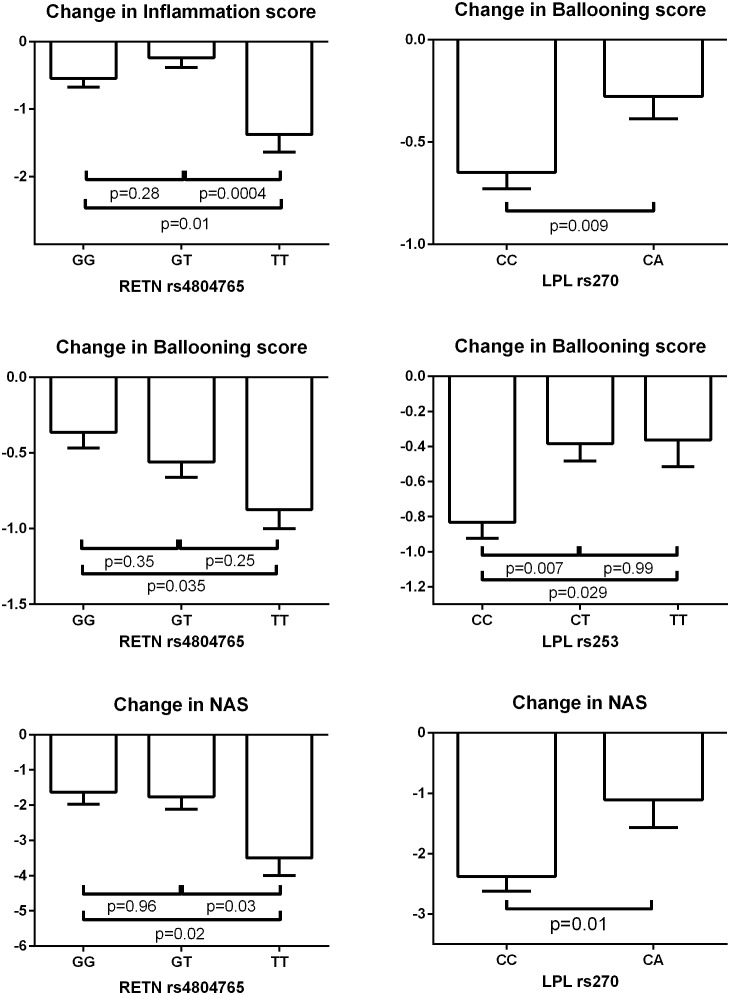
Change in biopsy scores based on genotypes of SNPs identified by meta-analyses. The bar columns and error bars represent the means and the standard errors of the means respectively. The shown *p*-values are adjusted for multiple comparisons by Tukey’s method.

**FIGURE 4:**
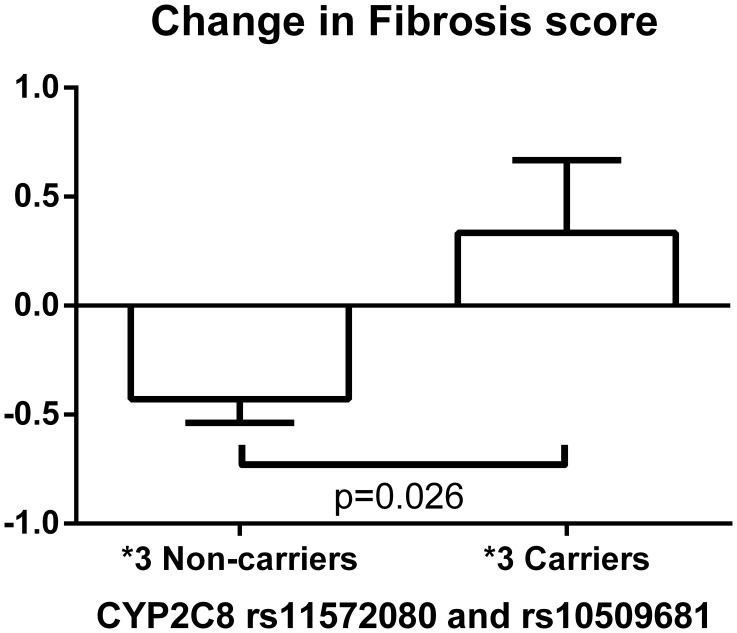
Change in fibrosis score based on *CYP2C8^∗^3* haplotype (rs11572080 and rs10509681) carrier status. The bar column and error bar represent the mean and the standard error of the mean respectively.

**Figure 3** depicts significant changes in histology (inflammation, ballooning or NAS) based on genotypes of the SNPs identified by the meta-analyses. **Figure 4** represents the top SNP associated with change in fibrosis – *CYP2C8* rs11572080. When an analysis was conducted based on *CYP2C8^∗^3* allele designation (carriers vs. non-carriers) and adjusted for age, gender, and baseline fibrosis score, the *CYP2C8^∗^3* allele was similarly associated with smaller change in fibrosis (β = 0.61, *p* = 0.0056, **Figure 4**).

When each race/ethnicity group was analyzed separately after adjusting for age, gender, and baseline histology score, *PPARG* rs17817276 G minor allele was associated with worse improvement in fibrosis score among Hispanic population, meeting Bonferroni-corrected significance (β = 0.85, *p* = 0.0002); Hispanic participants with *PPARG* rs17817276 variant allele had worse change in fibrosis score after pioglitazone therapy. During sensitivity analysis, the *ADORA1* rs903361 showed significant association with change in ballooning among Caucasian and Hispanic populations (β = −0.33, *p* = 0.00048, Supplementary Figure S1). No other statistically significant SNPs were detected in individual race/ethnicity groups.

Supplementary Figures S2–S6 include forest plots of the nominally significant SNPs for changes in steatosis, inflammation, ballooning, fibrosis, and NAS respectively.

## Discussion

This is the first study to investigate genetic associations with pioglitazone histologic response in patients with NASH. We examined multiple SNPs selected on the basis of reported associations with TZD response in patients with diabetes, and we replicated similar effects of polymorphisms in patients treated with pioglitazone for NASH. We also identified novel association of several SNPs with pioglitazone response in NASH patients. The novel findings from this study can be summarized as follows: (1) *PPARG* rs4135275 was the top SNP associated with the primary outcome; (2) *PPARG* rs17817276 was associated with change in fibrosis among Hispanics; (3) *LPL* rs253, *LPL* rs270, *LPL* rs10099160, *LPL* rs13266204, and *RETN* rs4804765 were SNPs consistently identified in association with plasma AST/ALT and histologic changes; and (4) the genetic response score based on the five SNPs for the primary outcome showed significant predictive value to this end. Taken together, this is a first step toward developing a genetic-based precision medicine approach. Moreover, it holds potential for tailoring TZD treatment to those patients most likely to benefit while sparing from drug exposure those least likely to respond.

As noted, the purpose of this study was to identify SNPs that associate with the histologic response to pioglitazone treatment in patients with NASH. The SNPs assessed have been linked to pioglitazone or TZD response in other populations (e.g., Type 2 diabetes) and for other outcomes (e.g., Hemoglobin A1c, fasting glucose, weight gain). In this study, the top SNP associated with the achievement of the primary outcome in the pioglitazone-treated patients with NASH was rs4135275, an intronic SNP in *PPARG* (*p* = 0.019) (UCSC Genome Browser, 2018). The *PPARG* gene codes for PPAR-γ, the drug target of pioglitazone, which suggests the genetic polymorphism may contribute to variability in drug response (Gene Database, 2018). Another intronic variant of *PPARG*, rs17817276 G minor allele, was also significantly associated with less improvement in fibrosis score among Hispanic participants (*p* = 0.0002). The two non-synonymous SNPs, *CYP2C8* rs11572080 and rs10509681, designating the *^∗^3* allele were previously linked to lower pioglitazone exposure (Tornio et al., 2008; Aquilante et al., 2013; Kawaguchi-Suzuki and Frye, 2013), which we have shown is an important determinant of response in patients with NASH (Kawaguchi-Suzuki et al., 2017). In this study, the *CYP2C8^∗^3* allele carriers exhibited less improvement in fibrosis score (*p* = 0.006). Because fibrosis is the single most important histologic feature that predicts future end-stage liver disease, this work may help in the future to identify those patients more likely to respond to pioglitazone therapy. Clearly, larger numbers of patients should be studied to confirm this provocative finding. Another gene associated with changes in fibrosis was *LPIN1*, a gene that encodes a magnesium-ion-dependent, phosphatidic acid phosphohydrolase involved in the penultimate step in triglyceride synthesis (Gene Database, 2018). *LPIN1* is required for adipocyte differentiation and, as a coactivator of PPAR, modulates expression of other genes necessary for lipid metabolism in hepatocytes and adipocytes. *LPIN1* is also a known susceptibility gene for metabolic syndrome and type 2 diabetes. A previous study demonstrated better response to rosiglitazone with the *LPIN1* rs10192566 G allele in type 2 diabetic patients (Kang et al., 2008). Although a later study did not replicate this finding, the baseline A1c among rs10192566 minor allele carriers was statistically lower than that among patients with the homozygous wild type (Stage et al., 2013). In this study, participants with the *LPIN1* rs10192566 G minor allele had better improvement in the fibrosis score when treated with pioglitazone for NASH (*p* = 0.0095), matching the direction of the genetic effect with the previous finding.

One of the SNPs showing consistent association with biopsy results was *ABCA1* rs2230806, which was also the top SNP associated with change in steatosis. The *ABCA1* gene codes for an ATP-binding cassette transporter that works as a cholesterol efflux pump in the cellular lipid removal pathway and is crucial for the catabolism of high-density lipoproteins (Gene Database, 2018). Interestingly, *ABCA1* rs2230806 is a functional, non-synonymous SNP that encodes a substitution of lysine for arginine at position 219 (Wang et al., 2008). Previously in type 2 diabetic patients, the wild type homozygotes had better response to rosiglitazone in terms of improvement in insulin sensitivity (Wang et al., 2008). In this study, the wild type homozygotes had higher chance of NASH resolution (*p* = 0.019) and better changes in steatosis (*p* = 0.0087), ballooning (*p* = 0.031), and NAS (*p* = 0.0086) than the minor allele carriers, showing the same direction of the genetic effect in patients treated for NAFLD with pioglitazone.

The top SNP identified for resolution of NASH was *KCNQ1* rs2237895. *KCNQ1* encodes the pore-forming subunit of the voltage-gated K^+^ channel located not only in cardiac muscles but also in islet cells, the liver, and skeletal muscles; it is considered a susceptibility gene for type 2 diabetes (Finsterer and Stöllberger, 2004; Unoki et al., 2008; Yasuda et al., 2008; Yu et al., 2011). In an earlier study, *KCNQ1* rs2237895 C minor allele carriers demonstrated less favorable response to rosiglitazone therapy in patients treated for type 2 diabetes (Yu et al., 2011). The patients with diabetes carrying the *KCNQ1* rs2237895 C allele had a smaller change in both fasting insulin level and HOMA-IR (Yu et al., 2011). In the current study, the *KCNQ1* rs2237895 C allele was associated with a smaller likelihood of achieving resolution of NASH (*p* = 0.012) and worse change in ballooning (*p* = 0.0084), consistent with the direction of the polymorphic effect with the earlier findings.

Multiple SNPs located in the *LPL* gene associated with pioglitazone response in this study. The SNPs with consistent association with biopsy results and/or biomarker levels were: *LPL* rs253, *LPL* rs270, *LPL* rs10099160, and *LPL* rs13266204. *LPL* rs253 variant carriers had a smaller chance of achieving the primary outcome (*p* = 0.022) and worse change in ballooning (*p* = 0.0057). *LPL* rs270 variant allele was associated with smaller chance of achieving the primary outcome (*p* = 0.027) and NASH resolution (*p* = 0.019) and worse changes in ballooning (*p* = 0.0048) and NAS (*p* = 0.0045), as well as smaller reduction in plasma ALT levels (*p* = 0.01). The association of *LPL* rs10099160 with change in ALT met Bonferroni-corrected significance (*p* = 0.0005); this SNP was associated with the primary outcome (*p* = 0.026) and change in NAS (*p* = 0.018). Participants with *LPL* rs13266204 variant allele had worse change in ballooning (*p* = 0.011) and NAS (*p* = 0.0067). The *LPL* gene codes for lipoprotein lipase, which is expressed in adipocytes, heart, and muscle; the rs253, rs270, rs10099160, and rs13266204 SNPs are intronic variants (UCSC Genome Browser, 2018). Lipoprotein lipase is a known triglyceride hydrolase and also works as a ligand and bridging factor for the uptake of receptor-mediated lipoproteins. *LPL* may be a promising gene for future studies to further understand the involvement of lipoprotein lipase in the treatment of NAFLD.

The combined effect of identified polymorphisms was examined using the four *LPL* SNPs with the top *PPARG* SNP for the primary outcome to calculate a genetic response score for individual participants (Supplementary Table S2). The calculated genetic response scores showed significant impact on achievement of the primary outcome (*p* = 0.0015). That a composite genetic score associated with achievement of the primary outcome is not unexpected for a complex phenotype such as NAFLD; multiple genetic factors likely contribute to the response to pioglitazone. These data suggest that a genetic response score might be built to inform shared treatment decisions. For example, patients having a score in the lower quartile (or below some validated cutoff value) may decide not to undergo 18-months of treatment with pioglitazone because the likelihood of a positive outcome is low. The use of such a genetic response score will require validation in a larger sample size.

*RETN* rs4804765 was another SNP consistently associated with biopsy results. *RETN* rs4804765 variant carriers showed less likelihood of obtaining the primary outcome (*p* = 0.03) and worse change in steatosis (*p* = 0.033), inflammation (*p* = 0.0078), ballooning (*p* = 0.034), and NAS (*p* = 0.0028). *RETN* gene codes for resistin; rs4804765 is a SNP located on the 3′ side of the gene (Gene Database, 2018). The mouse homolog of this protein is secreted by adipocytes and is a hormone potentially linking obesity to type 2 diabetes. In the Framingham Offspring Study, a strong association of *RETN* rs4804765 with resistin levels was observed (Hivert et al., 2009). The SNP may affect the expression level of the hormone possibly leading to difference in the phenotype.

*ADORA1* rs903361 is a SNP located 6.1 kb upstream of *ADORA1* gene encoding the adenosine A1 receptor known to inhibit adenylyl cyclase. The type A1 receptor is dominant in adipocytes and shown to mediate the inhibition of lipolysis (Gharibi et al., 2012). Previously, patients with type 2 diabetes were shown to have higher body mass index after pioglitazone therapy when they carried the minor allele, suggesting higher sensitivity to the drug with the presence of this particular polymorphism (Ruaño et al., 2009). In the current study, participants with the *ADORA1* rs903361 minor allele had better response to pioglitazone therapy and were more likely to resolve NASH, confirming the same direction of the genetic effect. Among the top SNPs, the association of the *ADORA1* rs903361 minor G allele with resolution of NASH was significant by the Cochran Armitage trend test as well (*p* = 0.0005; **Figure 3**). In addition, the *ADORA1* rs903361 showed significant association with change in ballooning among Caucasian and Hispanic populations (*p* = 0.0005).

*ADIPOQ* gene is exclusively expressed in adipose tissues and encodes adiponectin which has roles in various metabolic and hormonal processes. *ADIPOQ* rs266729 and rs182052 SNPs were associated with changes in fibrosis in this study; participants with the variant allele had better improvement in the fibrosis score (*p* = 0.02 and *p* = 0.04, respectively). Previously, *ADIPOQ* rs266729 and rs182052 minor alleles were associated with better A1c response in patients with type 2 diabetes treated with pioglitazone (Li et al., 2008). The direction of the effect by *ADIPOQ* rs266729 and rs182052 minor alleles was the same in this current study population with NAFLD. Hence, the SNPs previously associated with TZD response among diabetic patients for which we were able to identify similar effects in our study population with NASH in terms of liver biopsy findings were: *ABCA1* rs2230806, *ADIPOQ* rs266729 and rs182052, *ADORA1* rs903361, *CYP2C8* rs11572080 and rs10509681, *KCNQ1* rs2237895, and *LPIN1* rs10192566.

This study had several limitations that should be noted. The biggest limitation was the relatively small sample size. Although participants were genotyped only for polymorphisms with relatively high minor allele frequencies, the percentage of participants carrying the minor allele turned out to be small for some SNPs, lowering the power of the study. This was considered a likely reason for the difficulty of meeting Bonferroni-corrected significance. In addition, the largest race/ethnicity group in this study was Hispanic; only six participants were categorized in the “other” race/ethnicity group. In this study, the MAF for significant SNPs and the responses were similar between Caucasians and Hispanics (Supplementary Table S3) but not significant in Caucasians due to the small number of Caucasians. Since meta-analysis was conducted to consider for any possible population stratification, the forest plots should be examined to interpret the results carefully. Another constraint of this study was the limited number of SNPs we were able to include on our customized chips. In addition to the limited number of SNPs, due to the availability of the probes, the 60 SNPs were not an inclusive list of SNPs previously identified among diabetic patients or tagged by HaploView. Furthermore, many of the identified SNPs in association with the liver biopsy findings were located in an intron. It is possible for the intronic variants to affect the expression of the gene, leading to the difference in the phenotype. However, it is also possible that the identified SNP is in linkage disequilibrium with other functional SNPs not genotyped in this study. Further studies are warranted to confirm the identified effect of the polymorphisms in a larger study cohort.

## Conclusion

This is the first study to identify key genetic effects on the inter-individual variability in response to pioglitazone treatment among patients with NASH. A strength of the study was that the candidate genes were selected based on the drug response pathway and/or the pathophysiology of NAFLD making the biologic plausibility easier to be identified. In order to translate study data and move on to clinical applications, replication of findings are crucial, especially for pharmacogenomic findings. This current study was able to replicate previously identified functional SNPs among diabetic patients (Kawaguchi-Suzuki and Frye, 2013) in an independent cohort of patients with NASH. The same direction of the genetic effect was demonstrated and described above. Thus, the current study was able to confirm previously found genetic effects in this unique patient population with NASH and to further detect novel genetic markers, which may ultimately help identify patients who may benefit from pioglitazone treatment for NASH.

## Author Contributions

MK-S, FB, KC, YG, TL, and RF analyzed and interpreted the data and drafted the manuscript. All authors were involved in conceptualizing and designing the study, acquiring data, critically revising the manuscript for important intellectual content, and/or obtaining funding.

## Conflict of Interest Statement

The authors declare that the research was conducted in the absence of any commercial or financial relationships that could be construed as a potential conflict of interest.

## Abbreviations

ALT: alanine aminotransferase
AST: aspartate aminotransferase
CK-18: cytokeratin-18
CYP: cytochrome P450
DNA: deoxyribonucleic acid
MAF: minor allele frequency
NAFLD: non-alcoholic fatty liver disease
NAS: NAFLD activity score
NASH: non-alcoholic steatohepatitis
OR: odds ratio
PPAR-γ: peroxisome proliferator-activated receptor-γ
SNP: single nucleotide polymorphism
TZD: thiazolidinedione
95% CI: 95% confidence interval
