# Supplementary material for: A Genetic Score Associates With Pioglitazone Response in Patients With Non-alcoholic Steatohepatitis
Source: Front Pharmacol. 2018 Jul 17;9:752. doi: 10.3389/fphar.2018.00752 (PMC6056641; doi:10.3389/fphar.2018.00752)
Supplement: Supplementary file 1 [file Image_1.PDF]

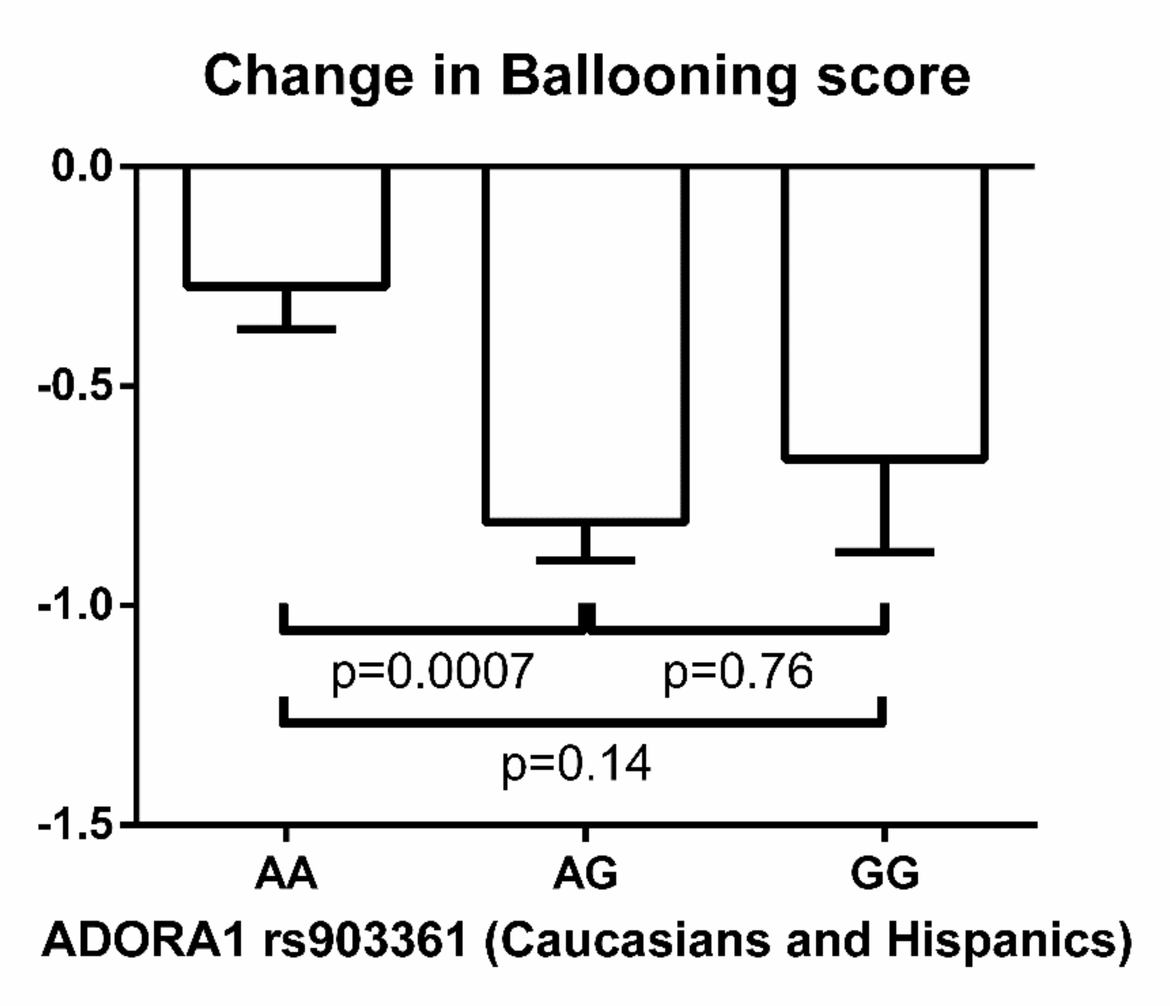

Figure S1. Change in ballooning score based on ADORA1 rs903361 genotypes. Only Caucasians and Hispanics are included in this analysis. The bar columns and error bars represent the mean and the standard error of the mean respectively. The shown p-values are adjusted for multiple comparisons by Tukey’s method.
