# Supplementary material for: A Genetic Score Associates With Pioglitazone Response in Patients With Non-alcoholic Steatohepatitis
Source: Front Pharmacol. 2018 Jul 17;9:752. doi: 10.3389/fphar.2018.00752 (PMC6056641; doi:10.3389/fphar.2018.00752)
Supplement: Supplementary file 6 [file Image_6.PDF]

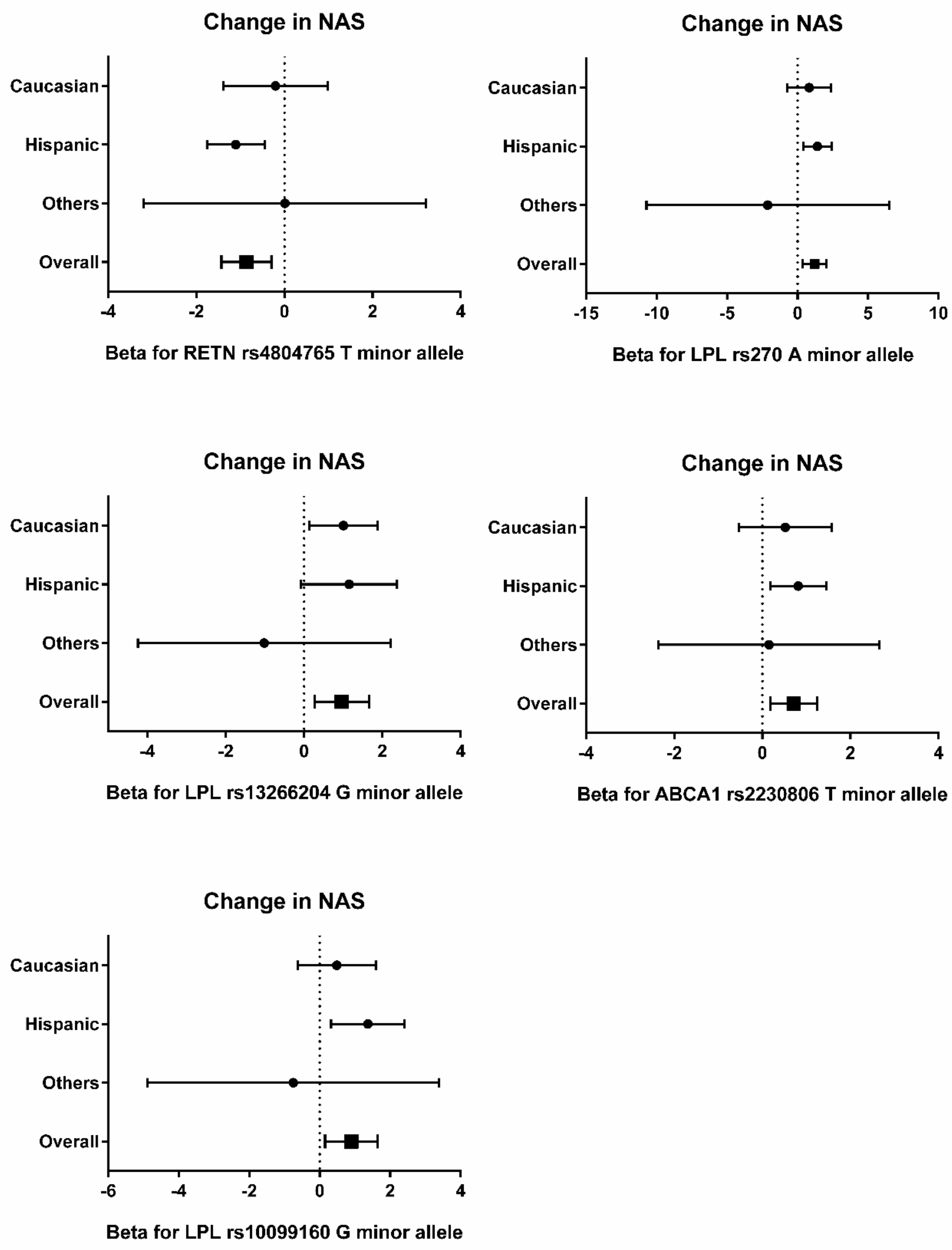

Figure S6. Forest plots of the top SNPs for NAS. The analysis is adjusted for age, gender, and baseline NAS.
