# Supplementary material for: A Genetic Score Associates With Pioglitazone Response in Patients With Non-alcoholic Steatohepatitis
Source: Front Pharmacol. 2018 Jul 17;9:752. doi: 10.3389/fphar.2018.00752 (PMC6056641; doi:10.3389/fphar.2018.00752)
Supplement: Supplementary file 7 [file Table_1.DOCX]

Table S1. List of the 60 SNPs genotyped.

| **Gene** | **SNP** |
| --- | --- |
| CYP2C8 | rs11572080 |
|  | rs10509681 |
|  | rs11572103 |
|  | rs1058930 |
| PPARG | rs1801282 |
|  | rs12629240 |
|  | rs709151 |
|  | rs2028759 |
|  | rs6809832 |
|  | rs6794024 |
|  | rs17817276 |
|  | rs1151996 |
|  | rs7626560 |
|  | rs17793693 |
|  | rs4135247 |
|  | rs4135275 |
|  | rs1152003 |
|  | rs2972164 |
|  | rs12497191 |
|  | rs9833097 |
| PPARGC1A | rs8192678 |
| PTPRD | rs17584499 |
| ADIPOQ | rs2241766 |
|  | rs266729 |
|  | rs182052 |
|  | rs1501299 |
|  | rs3821799 |
|  | rs6773957 |
|  | rs16861194 |
| RETN | rs1862513 |
|  | rs4804765 |
|  | rs3745367 |
|  | rs7408174 |
| LPL | rs328 |
|  | rs1534649 |
|  | rs13266204 |
|  | rs253 |
|  | rs269 |
|  | rs270 |
|  | rs297 |
|  | rs326 |
|  | rs327 |
|  | rs10099160 |
|  | rs4922115 |
|  | rs9644636 |
|  | rs2197089 |
| ADORA1 | rs903361 |
| LPIN1 | rs10192566 |
| KCNQ1 | rs2237892 |
|  | rs2237895 |
|  | rs2237897 |
| UCP2 | rs659366 |
| ADRB3 | rs4994 |
| LEP | rs7799039 |
| TNF | rs1800629 |
| PLIN1 | rs894160 |
| ABCA1 | rs2230806 |
|  | rs2230808 |
|  | rs2066714 |
| SLC30A8 | rs13266634 |
