# Supplementary material for: A Genetic Score Associates With Pioglitazone Response in Patients With Non-alcoholic Steatohepatitis
Source: Front Pharmacol. 2018 Jul 17;9:752. doi: 10.3389/fphar.2018.00752 (PMC6056641; doi:10.3389/fphar.2018.00752)
Supplement: Supplementary file 8 [file Table_2.DOCX]

Table S2. Calculation of SNP summarized score.

| SNP | Genotype | Score |
| --- | --- | --- |
| PPARG rs4135275 | AA | 0 |
|  | AG | 1 |
|  | GG | 2 |
| LPL rs253 | CC | 2 |
|  | CT | 1 |
|  | TT | 0 |
| LPL rs10099160 | TT | 2 |
|  | TG | 1 |
|  | GG | 0 |
| LPL rs270 | CC | 2 |
|  | CA | 1 |
|  | AA | 0 |
| LPL rs2197089 | AA | 0 |
|  | AG | 1 |
|  | GG | 2 |
| SNP summarized score = Sum of the scores above | | |
