# Supplementary material for: A Genetic Score Associates With Pioglitazone Response in Patients With Non-alcoholic Steatohepatitis
Source: Front Pharmacol. 2018 Jul 17;9:752. doi: 10.3389/fphar.2018.00752 (PMC6056641; doi:10.3389/fphar.2018.00752)
Supplement: Supplementary file 9 [file Table_3.DOCX]

Table S3. Minor allele frequencies by each race/ethnicity for SNPs in Table 3.

| SNP | Allele | Frequency (%) | | |
| --- | --- | --- | --- | --- |
|  |  | Caucasian | Hispanic | Others |
| PPARG rs4135275 | G | 0.25 | 0.25 | 0.07 |
| LPL rs253 | T | 0.34 | 0.43 | 0.64 |
| LPL rs10099160 | G | 0.28 | 0.21 | 0.14 |
| LPL rs270 | A | 0.19 | 0.16 | 0.14 |
| LPL rs2197089 | G | 0.63 | 0.45 | 0.36 |
| RETN rs4804765 | T | 0.31 | 0.43 | 0.29 |
| LPL rs13266204 | G | 0.22 | 0.10 | 0.29 |
| ABCA1 rs2230806 | T | 0.31 | 0.39 | 0.57 |
